#  Prevalent, protective, and convergent IgG recognition of SARS-CoV-2 non-RBD spike epitopes

**DOI:** 10.1126/science.abg5268

**Published:** 2021-05-04

**Authors:** William N. Voss, Yixuan J. Hou, Nicole V. Johnson, George Delidakis, Jin Eyun Kim, Kamyab Javanmardi, Andrew P. Horton, Foteini Bartzoka, Chelsea J. Paresi, Yuri Tanno, Chia-Wei Chou, Shawn A. Abbasi, Whitney Pickens, Katia George, Daniel R. Boutz, Dalton M. Towers, Jonathan R. McDaniel, Daniel Billick, Jule Goike, Lori Rowe, Dhwani Batra, Jan Pohl, Justin Lee, Shivaprakash Gangappa, Suryaprakash Sambhara, Michelle Gadush, Nianshuang Wang, Maria D. Person, Brent L. Iverson, Jimmy D. Gollihar, John Dye, Andrew Herbert, Ilya J. Finkelstein, Ralph S. Baric, Jason S. McLellan, George Georgiou, Jason J. Lavinder, Gregory C. Ippolito

**Affiliations:** 1 Department of Molecular Biosciences, The University of Texas at Austin, Austin, TX, USA.; 2 Department of Epidemiology, University of North Carolina at Chapel Hill, Chapel Hill, NC, USA.; 3 Department of Chemical Engineering, The University of Texas at Austin, Austin, TX, USA.; 4 Department of Biomedical Engineering, The University of Texas at Austin, Austin, TX, USA.; 5 Department of Chemistry, The University of Texas at Austin, Austin, TX, USA.; 6 U.S. Army Medical Research Institute of Infectious Diseases, Frederick, MD, USA.; 7 CCDC Army Research Laboratory-South, The University of Texas at Austin, Austin, TX, USA.; 8 Biomedicine Design, Pfizer, Cambridge, MA, USA.; 9 Biotechnology Core Facility Branch, Division of Scientific Resources, National Center for Emerging and Zoonotic Infectious Diseases, Centers for Disease Control and Prevention, Atlanta, GA, USA.; 10 Tulane National Primate Research Center Department of Microbiology 18703 Three Rivers Road Covington, LA, USA.; 11 Immunology and Pathogenesis Branch, Influenza Division, National Center for Immunization and Respiratory Diseases, Centers for Disease Control and Prevention, Atlanta, GA, USA.; 12 Center for Biomedical Research Support, The University of Texas at Austin, Austin, TX, USA.; 13 Department of Pathology and Genomic Medicine, Houston Methodist Research Institute, Houston Methodist Hospital, Houston, TX, USA.; 14 Department of Microbiology and Immunology, University of North Carolina at Chapel Hill, Chapel Hill, NC, USA.; 15 Department of Oncology, Dell Medical School, The University of Texas at Austin, Austin, TX, USA.

## Abstract

The molecular composition and binding epitopes of the immunoglobulin G (IgG) antibodies that circulate in blood plasma following SARS-CoV-2 infection are unknown. Proteomic deconvolution of the IgG repertoire to the spike glycoprotein in convalescent subjects revealed that the response is directed predominantly (>80%) against epitopes residing outside the receptor-binding domain (RBD). In one subject, just four IgG lineages accounted for 93.5% of the response, including an N-terminal domain (NTD)-directed antibody that was protective against lethal viral challenge. Genetic, structural, and functional characterization of a multi-donor class of “public” antibodies revealed an NTD epitope that is recurrently mutated among emerging SARS-CoV-2 variants of concern. These data show that “public” NTD-directed and other non-RBD plasma antibodies are prevalent and have implications for SARS-CoV-2 protection and antibody escape.

The SARS-CoV-2 spike ectodomain (S-ECD) folds into a multidomain architecture (*1*, *2*) and includes the RBD, which is essential for viral infectivity, and the structurally adjacent NTD, which plays an uncertain role. Humoral immunity to the spike (S) surface glycoprotein can correlate with protection, (*3*) and it is the primary antigenic target for most vaccines and monoclonal antibodies (mAbs). That the B cell repertoire can recognize multiple spike epitopes is supported by extensive single-cell cloning campaigns (*4*–*9*). However, the identity, abundance, and clonality of the IgG plasma antibody repertoire and the epitopes it may target are not known (*10*–*12*). Divergence between the two repertoires is biologically plausible (*13*–*17*) and the evidence in COVID-19 includes a paradoxical disconnect between virus-neutralizing IgG titers and RBD-specific B cell immunity (*6*, *11*, *18*, *19*).

To analyze the IgG repertoire, blood was collected during early convalescence from four seroconverted study subjects (P1–P4) who experienced mild COVID-19 disease that manifested with plasma virus-neutralization titers in the lowest quartile (P1 and P3), the second highest quartile (P2), or the highest quartile (P4) compared to a larger cohort (table S1 and fig. S1). The lineage composition and relative abundance of constituent IgG antibodies comprising the plasma response to either intact stabilized S-ECD (S-2P (*1*)) or RBD was determined using the Ig-Seq pipeline (*13*, *14*, *20*) that integrates analytical proteomics of affinity purified IgG fractions with peripheral B cell antibody variable region repertoires (BCR-Seq).

IgG lineages detected by Ig-Seq in the S-ECD fraction but absent from the RBD fraction were deemed to be reactive with spike epitopes outside the RBD. In subject P3, we detected six IgG lineages that bound to S-ECD (Fig. 1A). Four of these (Lin.1 to Lin.4) accounted for 93.5% abundance of the total plasma IgG S-ECD response and exhibited extensive intralineage diversity (fig. S2) indicative of clonal expansion and selection. Notably, the top three lineages (Lin.1 to Lin.3; >85% abundance) all bound to non-RBD epitopes (S2 subunit or NTD). Bulk serology ELISAs recapitulated the Ig-Seq result and demonstrated similarly high levels of non-RBD-binding IgG (*P>*0.05) (Fig. 1B), confirming that RBD-binding plasma antibodies comprise only a minor proportion of all spike-binding IgG in naturally infected individuals (*21*). In all four subjects, the detected plasma IgG repertoire to S-ECD was oligoclonal, comprising only 6–22 lineages, with the top-ranked lineage comprising 15 to 50% total abundance. On average, 84% of the anti-S-ECD plasma IgG repertoire bound to epitopes outside the RBD (Fig. 1C), a finding consistent with data from single B cell analyses (*22*), and the most abundant plasma IgG lineage in all donors recognized a non-RBD epitope (Figs. 1A and 2A and fig. S3).

**Fig. 1:**
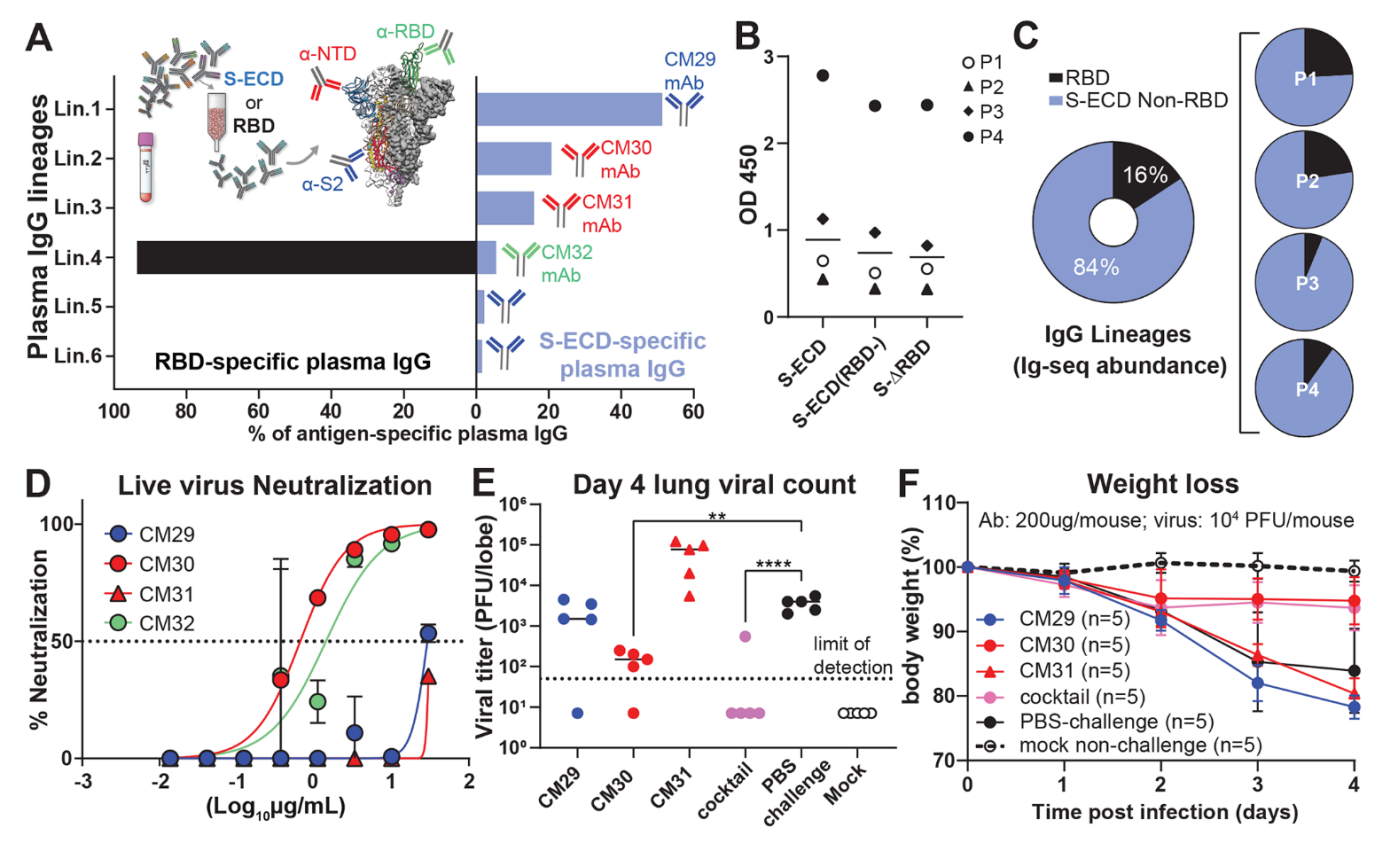
Most plasma IgG antibodies bind non-RBD spike epitopes such as the NTD. (**A**) Affinity-purification using spike S-ECD (*1*) or RBD for subject P3. Plasma IgG lineage identities, binding specificity, and relative abundance were mapped via Ig-seq proteomics (*14*), facilitating recombinant plasma mAb characterization; anti-RBD (green); anti-S2 (blue); anti-NTD (red). (**B**) IgG ELISA binding (1:150 plasma dilution) to S-ECD alone, or in the presence of 50 μg/ml of RBD (S-ECD(RBD-)) or S-∆RBD deletion mutant. (**C**) Quantitative Ig-seq determination of anti-RBD and non-RBD IgG mAb abundance in early convalescent plasmas across four subjects. (**D**) Authentic virus neutralization (in duplicate) of the four most abundant plasma IgGs (CM29, CM30, CM31, CM32) from plasma lineages Lin.1, Lin.2, Lin.3, Lin.4 in subject P3. (**E** and **F**) Prophylactic protection of 12-month-old BALB/c mice (n=5 per group) against lethal challenge with high dose (10^4^ PFU) mouse-adapted (MA10) SARS-CoV-2. Cocktail of non-RBD mAbs (200 μg per mouse) at 2:1:1 ratio reflecting their relative plasma abundance. **P<0.005; ****P<0.0001, determined by one-way ANOVA with Dunnett’s multiple comparisons test.

**Fig. 2:**
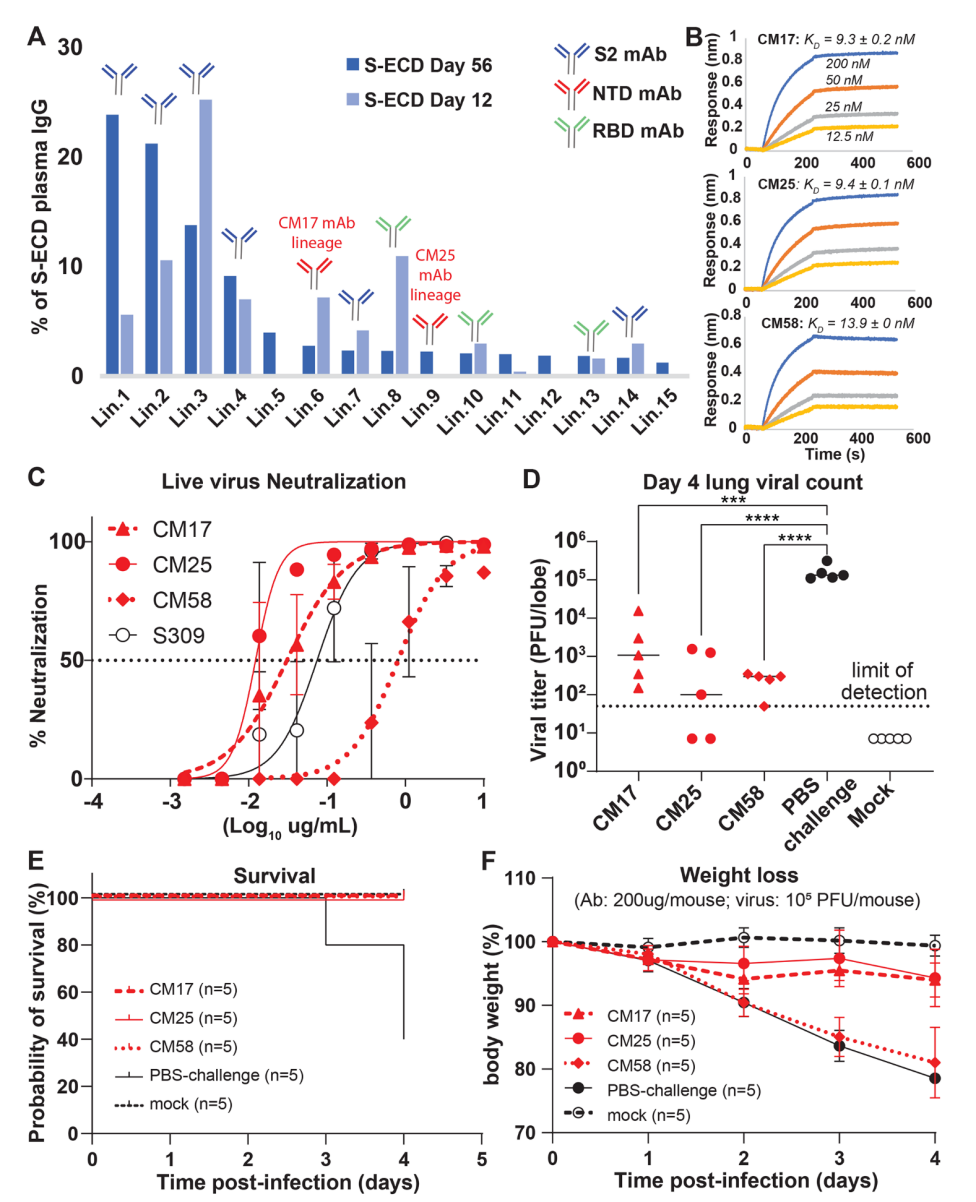
Protective spike NTD-targeting antibodies are prevalent in COVID-19 convalescent plasma. (**A**) Temporal Ig-seq dynamics of the anti-S-ECD IgG repertoire at days 12 and 56 post-symptom onset. (**B**) Biolayer interferometry (BLI) sensorgrams to S-ECD ligand of anti-NTD mAbs CM17, CM25 (subject P2), and CM58 (subject P4). (**C**) In vitro live virus neutralization (performed in duplicate). (**D-F**) In vivo prophylactic protection of 12-month-old BALB/c mice (n=5 per group) against high dose intranasal challenge (10^5^ PFU) of mouse-adapted (MA10) SARS-CoV-2. ***P<0.0007; ****P<0.0001, determined by one-way ANOVA with Dunnett’s multiple comparisons test.

Binding analysis of P3 mAbs CM29–CM32 representing the most expanded clones within each of lineages Lin.1 to Lin.4 showed that CM29 (Lin.1) recognizes the S2 subunit (K_D_ = 6.6 nM), CM30 and CM31 (Lin.2 and Lin.3 with K_D_ = 0.8 and 37.7 nM, respectively) were specific for the NTD, and CM32 (Lin.4) bound the RBD (K_D_ = 6.0 nM), as expected from the Ig-Seq differential affinity purifications (Fig. 1A and table S2). CM30 potently neutralized authentic SARS-CoV-2 in vitro (IC_50_ = 0.83 μg/ml), CM32 was slightly less potent (2.1 μg/ml), whereas CM29 and CM31 showed minimal neutralization activity (Fig. 1D).

We then determined the capacity of mAbs CM29–CM32, singly and in combination, to confer prophylactic protection in vivo to virus challenge using the MA10 mouse model of SARS-CoV-2 infection (*23*, *24*). Even though the RBD-directed mAb CM32 could neutralize authentic virus in vitro and had relatively high antibody-dependent cellular phagocytosis (ADCP) activity (fig. S4), it did not protect in vivo (fig. S5), possibly due to amino acid changes in the MA10 virus. Similarly, no protection was observed for the non-neutralizing S2-directed mAb CM29 or non-neutralizing NTD-directed mAb CM31. The neutralizing mAb CM30, derived from the top-ranking NTD-targeting IgG lineage (21% abundance), was the sole plasma antibody that conferred complete protection to MA10 viral challenge (Fig. 1, E and F, and fig. S5). Interestingly, administration of a cocktail comprising the top non-RBD plasma mAbs CM29–CM31 (>85% of the IgG plasma lineages to S-ECD; Fig. 1A) showed the most robust protection and lung viral titers below the limit of detection (LOD) in high viral load challenge (10^4^ PFU).

Subject P2, with ~10-fold higher neutralizing titer compared to subject P3 (fig. S1 and table S1), displayed a more polyclonal IgG response (Fig. 2A), with 12/15 lineages (>80% total abundance) in the anti-S-ECD repertoire recognizing non-RBD epitopes. Conspicuously, as with P3, the most abundant S-ECD-directed plasma antibodies target the S2 subunit, with the four topmost lineages (68% total abundance) binding to S2. MAbs CM25 and CM17, representative of two NTD-targeting lineages each comprising ~2.5% of the response at day 56 (Ig-Seq Lin.6 and Lin.9) (Fig. 2A), were both encoded by unmutated or near-germline IGHV1-24. We found an additional NTD-targeting unmutated IGHV1-24 plasma mAb (CM58) in subject P4. CM17, CM25 and CM58 bound S-ECD with similar single-digit nM affinity (Fig. 2B and table S2) and all three potently neutralized SARS-CoV-2 virus, with IC_50_ values of 0.01–0.81 μg/ml comparable to S309 anti-RBD control (*25*) (Fig. 2C, fig. S6, and table S2). For all three mAbs, pre-administration in the MA10 mouse model resulted in significantly reduced lung viral titers post-infection with 10^5^ PFU (Fig. 2D; *P*<0.001), resulting in 100% survival, compared to just 40% in the control group (Fig. 2E). CM17- and CM25-treated cohorts exhibited only minimal weight loss (Fig. 2F). Thus, IGHV1-24 is intrinsically suited for potent and protective targeting of the NTD.

B cell expression of IGHV1-24 in COVID-19 (~5 to 8%) (*5*, *7*, *26*) is ~10-fold higher than healthy individuals (0.4 to 0.8%) (*27*). Moreover, we could detect IGHV1-24 plasma antibodies only in S-ECD fractions (mean 3.7%), but not among anti-RBD IgGs (Fig. 3, A and B). Alignment of CM17, CM25, and CM58 with four neutralizing IGHV1-24 anti-NTD mAbs cloned from peripheral B cells [4A8 (*4*), 1-68 (*5*), 1-87 (*5*), COVA2-37 (*7*)] and an additional antibody [COV2-2199 (*8*)] identified a class of convergent V_H_ immune receptor sequences (Fig. 3C). In all cases, three glutamate (Glu) residues (Glu36, Glu59, and Glu80) located in complementarity-determining region (CDR)-H1, CDR-H2, and framework H3 (FWR-H3), respectively, as well as a phenylalanine (Phe) residue (Phe56) in CDR-H2, were invariably unmutated and are unique to the electronegative IGHV1-24 (pI=4.6). The convergent V_H_ genes paired promiscuously with six distinct light-chain V_L_ genes, yet CDR-H3 peptide lengths were restricted (14 or 21 amino acids) (Table S3). A “checkerboard” binding-competition experiment (Fig. 3D) indicated the presence of at least two epitope clusters on the NTD, including one targeted by all of the tested IGHV1-24 mAbs (4A8, CM25, CM17, CM58, and 1-68) and the IGHV3-11 mAb CM30. Another NTD epitope was identified by CM31 (IGHV2-5, 6.4% mutation), which overlapped with CM30 (IGHV3-11; 3.1% mutation), CM58, and 1-68 but did not compete with the other three IGHV1-24 NTD mAbs.

**Fig. 3:**
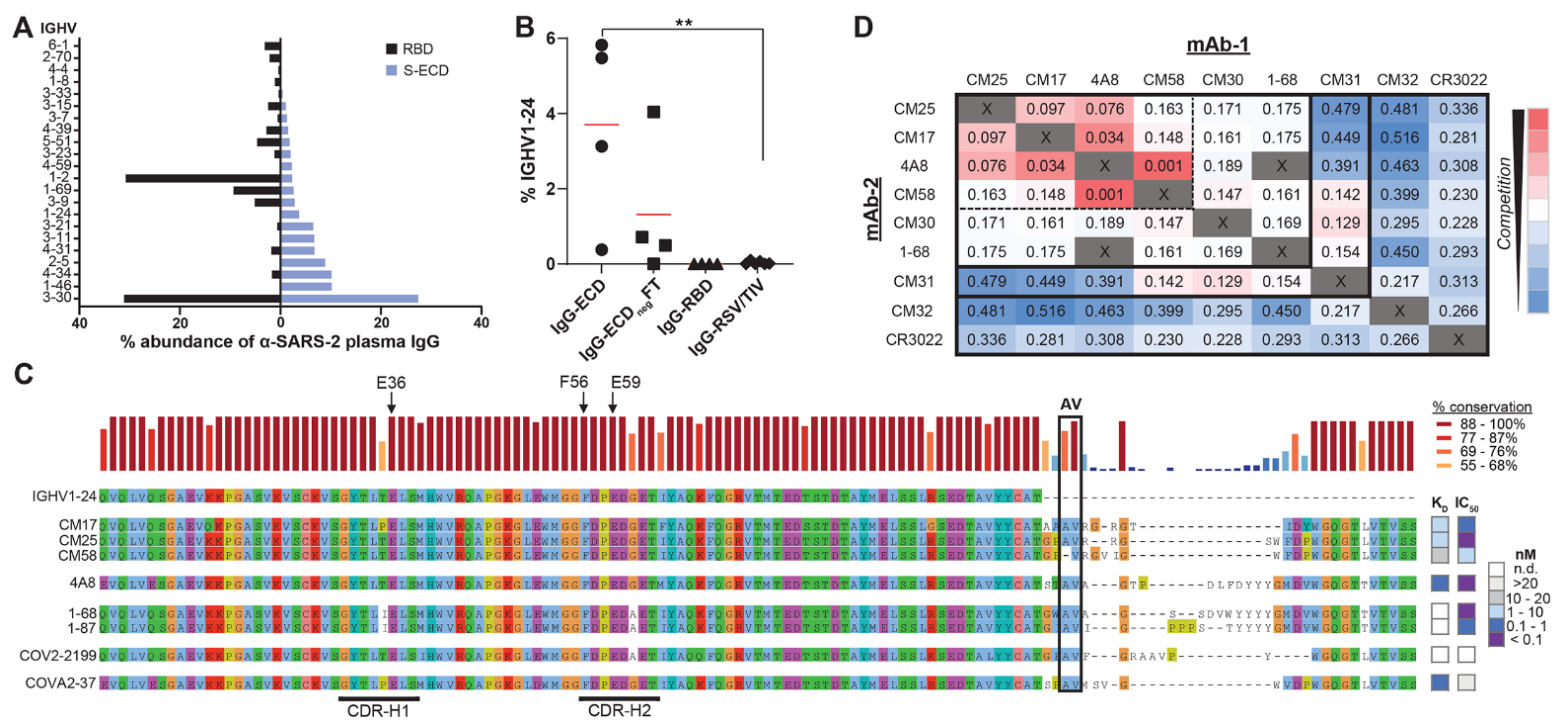
Genetic basis of a shared, or public, class of IGHV1-24 plasma antibodies targeting the spike NTD. (**A**) IGHV usage of plasma antibodies in all subjects (n=4). (**B**) Comparative IGHV1-24 usage of anti-S-ECD (IgG-ECD) and anti-RBD (IgG-RBD) plasma antibodies, or in depleted S-ECD affinity column flow through (IgG-ECD_neg_FT) in all subjects (n=4). IgG-RSV/TIV: IgG specific to respiratory syncytial virus (RSV) or trivalent influenza vaccine hemagglutinin HA1 (TIV) in healthy controls post-vaccination (n=6). **P<0.01, determined by Mann–Whitney *U* test. (**C**) Sequence alignment of IGHV1-24 neutralizing anti-NTD IgGs from plasma (CM17, CM25, and CM58) or from peripheral B cells (4A8 (*4*), 1-68 and 1-87 from a subject with ARDS (*5*), COV2-2199 (*13*), and COVA2-37 [mild disease subject]) (*7*). Arrows point to unique IGHV1-24 residues. Heatmap shows recombinant mAb affinity (K_D_) and live-virus neutralization (IC_50_) for individual antibodies. (**D**) Competitive BLI binding assay (“checkerboard competition”) of NTD-binding mAbs found in this study (CM17, CM25, CM58, CM30, and CM31) and others (4A8 and 1-68). RBD-binding mAbs CM32 and CR3022 included for comparison. Numbers refer to the shift, in nanometers, after second mAb binding to the preformed mAb–NTD complex. Dashed box drawn to highlight strong competition (<0.1 nm shift) among 4A8 and three IGHV1-24 mAbs examined in this study.

To better understand the IGHV1-24 interactions with the spike NTD, we determined a cryo-EM structure of CM25 Fabs bound to trimeric S-ECD (Fig. 4A and figs. S7 and S8). Focused refinement of the CM25-NTD interface resulted in a 3.5-Å reconstruction that revealed a heavy-chain–dominant mode of binding, with substantial contacts mediated by interactions between the three CDRs and the N3 and N5 loops of the NTD (Fig. 4B). The light chain contributes only 11% (86 Å^2^) of the total CM25 binding interface, mainly through a stacked hydrophobic interaction between CDR-L2 Tyr55 and Pro251 within the N5 loop. Unique germline IGHV1-24 residues contribute 20% (149 Å^2^) of the total binding interface. CDR-H1 interacts extensively through hydrogen bonds and contacts between hydrophobic residues, including a salt bridge formed between the conserved Glu36 residue and the N5 loop residue Arg246 (Fig. 4C). The common IGHV1-24 Phe56 residue in CDR-H2 forms a pi-cation interaction with Lys147 in the N3 loop (Fig. 4C). CM25 contains a 14-amino-acid CDR-H3 loop that contributes 35% (261 Å^2^) of the total interface, including the AV aliphatic motif found in all but one of the convergent IGHV1-24 NTD-binding mAbs. Ala109 and Val110 are buried at the interface in a binding pocket framed by the N3 and N5 loops. A comparison of CM25 with an extant structure of an IGHV1-24 NTD-binding antibody isolated by B cell cloning, 4A8 (*4*), revealed that the AV dipeptide interaction is structurally conserved, and the 21 amino-acid CDR-H3 of 4A8 extends along the outside of the NTD, contributing three additional contacts and 46% (415 Å^2^) of the total binding interface (Fig. 4D). Both structures show extensive contacts between the heavy chain of the Fabs and the NTD N3 and N5 loops. The Glu36-Arg246 salt bridge and an identical CDR-H2 contact between Phe56 and Lys147 are conserved in the 4A8-NTD interface.

**Fig. 4:**
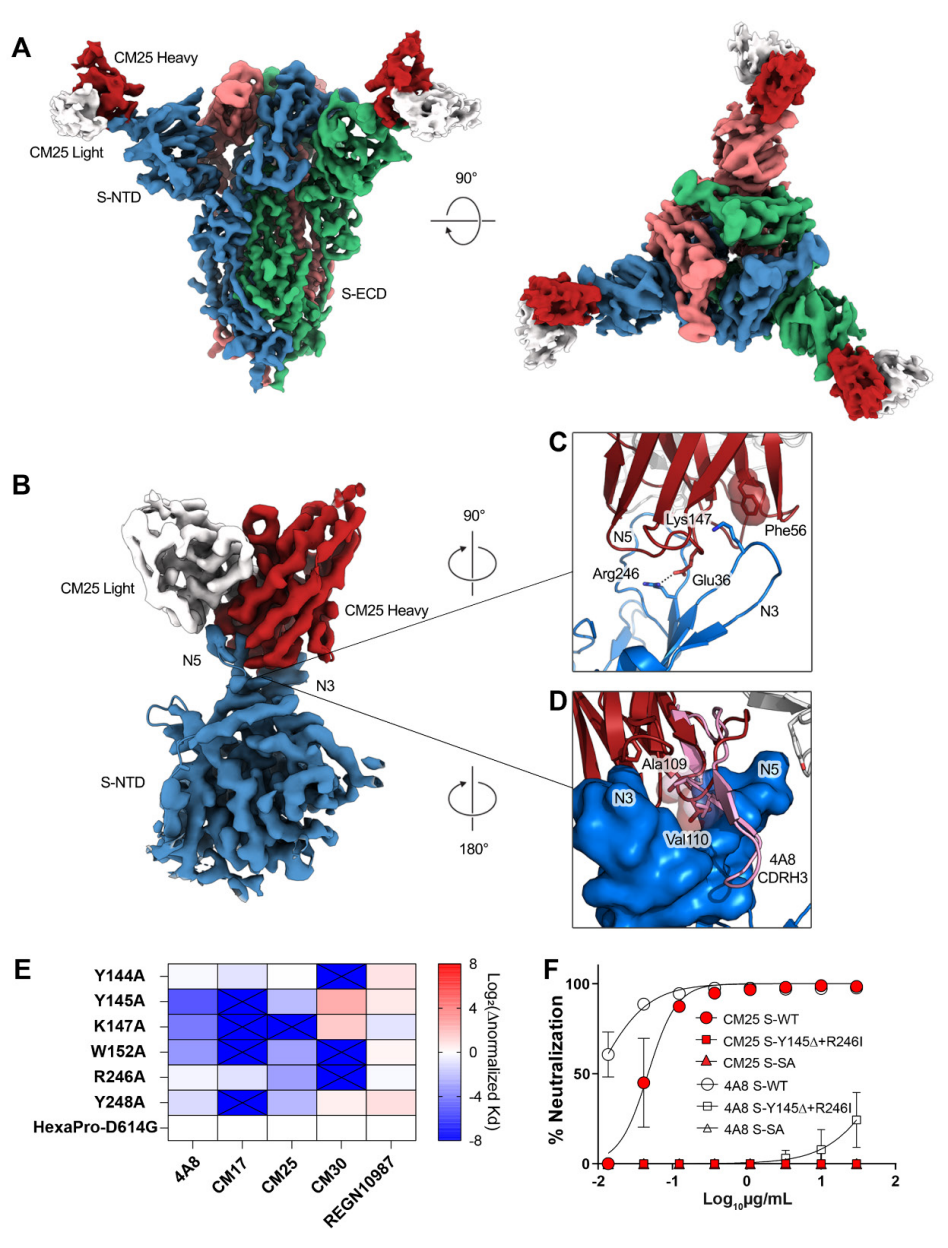
Structural basis of public IGHV1-24 plasma antibodies, NTD mutations, and antibody escape. (**A**) Side and top views of the structure of CM25 Fab bound to S-ECD shown as cryo-EM density. (**B**) Focused refinement density revealing a VH-dominant mode of binding, with substantial contacts mediated by interactions between the three CDRs and the N3 and N5 loops of the NTD. (**C**) CDR-H1 interaction includes a salt bridge formed between the uniquely encoded Glu36 residue and the N5 loop residue Arg246; Phe56 unique residue in CDR-H2 forms a pi-cation interaction with Lys147 in the N3 loop. (**D**) The AV dipeptide interaction with the N3 and N5 loops of the NTD is structurally conserved between mAbs CM25 (red) and 4A8 (pink). (**E**) Normalized shift (Log2) in binding K_D_, as measured by differential BLI affinities for single Ala mutants and parental D614G spike protein. (**F**) Authentic virus neutralization of CM25 and 4A8 against WT, double S-N3/N5 loop mutants, and South Africa (SA) B.1.351 viral variant.

SARS-CoV-2 variants of concern contain mutations in the NTD N3 and N5 loops, including Y144/Y145Δ and K147E (UK lineage B.1.1.7), W152C (California B.1.429), and 242-244Δ or R246I (South Africa B.1.351). Alanine substitutions at several of these positions ablated binding or reduced affinity more than fivefold by public IGHV1-24 antibodies as exemplified by 4A8, CM17, and CM25 (Fig. 4E and fig. S9), a result consistent with the CM25-NTD and 4A8-NTD structures. Additionally, we confirmed that an engineered N3-N5 double-mutant and native B.1.351 (*28*) both evade neutralization by mAbs CM25 and 4A8 (Fig. 4F). Thus, mutations in SARS-CoV-2 variants confer escape from public neutralizing anti-NTD antibodies.

In conclusion, we find that the convalescent plasma IgG response to SARS-CoV-2 is oligoclonal and directed overwhelmingly toward non-RBD epitopes in the S-ECD. This includes public, near-germline, and potently neutralizing antibodies against the NTD. The degree to which public anti-NTD antibodies contribute to protection is likely related to their relative levels in plasma, which can be dominant in some individuals. Our finding that mutations present in circulating SARS-CoV-2 variants can impair or ablate binding and neutralization by public anti-NTD antibodies may constitute a mechanism of viral escape in a subset of the population. Numerous other NTD mutations—which overlap with the structural epitope recognized by the public IGHV1-24 antibody class—have been described in additional circulating variants, in laboratory escape mutants, and in immunocompromised patients (*12*, *29*–*33*).

## Supplementary Materials

science.sciencemag.org/cgi/content/full/science.abg5268/DC1 Materials and Methods Figs. S1 to S9 Tables S1 to S5 References (*34*–*54*) MDAR Reproducibility Checklist
